# Management of corticosteroid-dependent eosinophilic interstitial nephritis: A case report

**DOI:** 10.1097/MD.0000000000028252

**Published:** 2021-12-17

**Authors:** Katsuyuki Tanabe, Natsumi Matsuoka-Uchiyama, Tomoyo Mifune, Chieko Kawakita, Hitoshi Sugiyama, Jun Wada

**Affiliations:** a Department of Nephrology, Rheumatology, Endocrinology and Metabolism, Okayama University Graduate School of Medicine, Dentistry and Pharmaceutical Sciences, Okayama, Japan; b Department of Human Resource Development of Dialysis Therapy for Kidney Disease, Okayama University Graduate School of Medicine, Dentistry and Pharmaceutical Sciences, Okayama, Japan.

**Keywords:** corticosteroid, drug-induced interstitial nephritis, eosinophils, mycophenolate mofetil, polypharmacy

## Abstract

**Introduction:**

Drug-induced acute interstitial nephritis (DI-AIN) is an important cause of acute kidney injury. In renal biopsy specimens, tubulitis with eosinophilic infiltration is suggestive of DI-AIN. Although corticosteroid therapy and discontinuation of the offending drug can improve renal dysfunction in most cases of DI-AIN, some patients experience AIN recurrence, leading to corticosteroid dependency. Corticosteroid-dependent eosinophilic interstitial nephritis presents a difficult dilemma in diagnosis and information regarding optimum management is limited.

**Patient concerns:**

A 25-year-old man, who received treatment with carbamazepine, zonisamide, valproate, and lacosamide for temporal lobe epilepsy, showed an increase in serum creatinine level from 0.98 to 1.29 mg/dL over a period of 6 months. Although he exhibited no symptoms, his serum creatinine level continued to increase to 1.74 mg/dL.

**Diagnosis:**

Renal biopsy revealed tubulitis and interstitial inflammatory infiltrates with eosinophils. Immunological and ophthalmological examinations showed no abnormal findings, and thus, his renal dysfunction was presumed to be caused by DI-AIN. Although oral prednisolone (PSL) administration (40 mg/d) and discontinuation of zonisamide immediately improved his renal function, AIN recurred 10 months later. The increase in PSL dose along with discontinuation of valproate and lacosamide improved renal function. However, 10 months later, recurrent AIN with eosinophilic infiltration was confirmed by further biopsy. The patient was therefore diagnosed with corticosteroid-dependent eosinophilic interstitial nephritis.

**Interventions:**

To prevent life-threatening epilepsy, carbamazepine could not be discontinued; hence, he was treated with an increased dose of PSL (60 mg/d) and 1500 mg/d of mycophenolate mofetil (MMF).

**Outcomes:**

MMF was well tolerated and PSL was successfully tapered to 5 mg/d; renal function stabilized over a 20-month period.

**Lessons:**

The presence of underdetermined autoimmune processes and difficulties in discontinuing the putative offending drug discontinuation are contributing factors to corticosteroid dependency in patients with eosinophilic interstitial nephritis. MMF may be beneficial in the management of corticosteroid-dependent eosinophilic interstitial nephritis by reducing the adverse effects related to high-dose and long-term corticosteroid use.

## 1 Introduction

Acute interstitial nephritis (AIN), an important cause of acute kidney injury, is histologically defined by an interstitial mononuclear cell infiltration with tubulitis characterized by inflammatory cells in the renal tubular wall. Drug-induced AIN (DI-AIN) accounts for approximately two-thirds of AIN cases.^[1]^ DI-AIN abruptly reduces renal function and is often associated with fever and/or eruption, whereas in some cases, renal dysfunction insidiously progresses without symptoms,^[2]^ suggesting the importance of histological diagnosis. Significant eosinophilic infiltration into the kidney is a characteristic finding of DI-AIN, which suggests an underlying allergic mechanism. However, a similar pathology can be observed in rare autoimmune disorders, thus diagnostic dilemmas are common in cases of eosinophilic interstitial nephritis.

The main therapeutic strategy for DI-AIN is to discontinue the offending drug; moreover, corticosteroid therapy is initiated in patients with moderate to severe renal injury. However, despite renal recovery after corticosteroid therapy, some patients experience AIN recurrence, thereby exposing them to repeated and long-term corticosteroid administration, which is known as corticosteroid dependency.^[3]^ Unfortunately, there is little information regarding the management of corticosteroid-dependent eosinophilic interstitial nephritis.

Herein, we present a case of corticosteroid-dependent AIN with renal eosinophilic infiltration in a patient with multiple anti-epileptic drug use; further, we discuss the factors contributing to corticosteroid dependency and potential strategies against recurrent eosinophilic interstitial nephritis. Written informed consent was obtained from the patient for publication of this case report.

## 2 Case presentation

**Figure 1:**
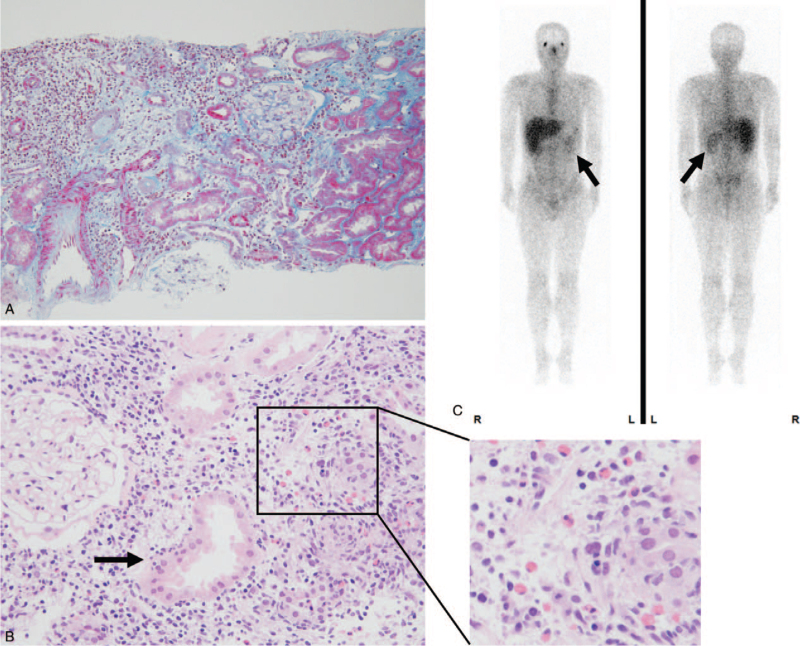
(A) Renal biopsy reveals massive inflammatory infiltration and marked tubular injury in the kidney (Masson-trichrome stain; original magnification ×100). (B) In the hematoxylin-eosin-stained specimen, inflammatory infiltration into tubular epithelial cells (tubulitis; arrow) is observed (original magnification ×200); an enlarged image (right) of the area marked by a box shows many eosinophils with acidophilic (reddish) cytoplasm in the interstitial infiltrates. (C) ^67^Ga scintigraphy reveals radioactive tracer uptake in both kidneys (arrows). No other abnormal uptake was observed.

A 25-year-old man was diagnosed with temporal lobe epilepsy 12 years ago, which was treated with carbamazepine and zonisamide. Subsequently, valproate and lacosamide were added 5 and 3 years ago, respectively, due to poor seizure control. During a regular visit, a neurologist noticed that the patient's serum creatinine level increased from 0.98 to 1.29 mg/dL over 6 months. As his serum creatinine level continued to increase to 1.74 mg/dL, he was referred to the nephrology department. He presented no symptoms, and physical examination revealed no abnormal findings. Laboratory analyses showed renal dysfunction, mildly elevated C-reactive protein levels, and increased levels of urinary markers for tubular injury (see Table 1). Renal biopsy was performed. Although the glomeruli were intact, there was marked interstitial lymphocytic and eosinophilic infiltration (Fig. 1A, B), suggestive of AIN. No immunoglobulin or complement deposition was observed. Since he had no other organ involvement (including uveitis) and no laboratory findings suggestive of autoimmune disorders such as sarcoidosis and Sjögren's syndrome, the AIN was ascribed to drug allergy. Based on a positive result of lymphocyte stimulation test (LST), zonisamide was discontinued and daily oral prednisolone (40 mg, 0.8 mg/kg) was started.

**Table 1:** Laboratory test results before corticosteroid therapy.

Laboratory test (unit)	Result	Reference range
Hemoglobin (g/dL)	13.1	13.7-16.8
White blood cells (/μL)	4,720	3,300–8,600
Eosinophils (/μL)	113	0-946
Platelets (×10^3^/μL)	286	158-348
Protein (g/dL)	7.5	6.6–8.1
Albumin (g/dL)	4.2	4.1–5.1
Blood urea nitrogen (mg/dL)	25.3	8.0–20.0
Creatinine (mg/dL)	1.74	0.65–1.07
Sodium (mmol/L)	139	138–145
Potassium (mmol/L)	3.8	3.6–4.8
Chloride (mmol/L)	105	101–108
Bicarbonate (mmol/L)	21.3	22.0–26.0
Calcium (mg/dL)	9.3	8.8–10.1
C-reactive protein (mg/dL)	1.77	<0.15
IgG (mg/dL)	1,404.6	861.0–1,470.0
IgG4 (mg/dL)	23.0	4.8–105.0
Anti-nuclear antibody	<1:40	<1:40
SS-A antibody (U/mL)	<0.50	<0.50
SS-B antibody (U/mL)	<0.50	<0.50
Angiotensin-converting enzyme (U/L)	6.6	8.3–21.4
Soluble IL-2 receptor (U/mL)	469	122–496
Cryoglobulin	Negative	Negative
MPO-ANCA (IU/mL)	<0.50	<3.50
PR3-ANCA (IU/mL)	<0.50	<2.50
Urinary N-acetyl-β-glucosaminidase (U/L)	48.0	0.3–15.0
Urinary β2-microglobulin (μg/mL)	21.51	<0.29
Urinary protein-to-creatinine ratio (g/g)	0.10	<0.15

**Figure 2:**
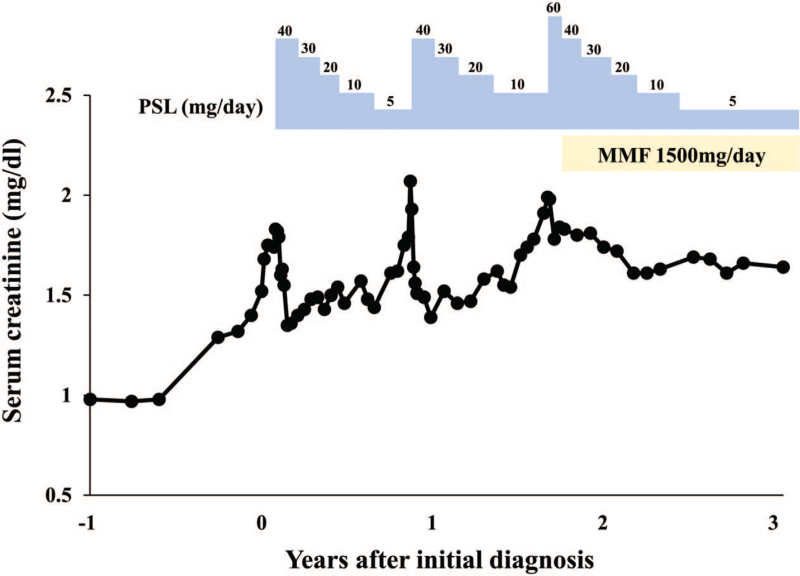
Clinical course of the patient with a trend of serum creatinine levels. Values on the blue steps indicate the daily dosage (mg) of prednisolone (PSL). A daily dose of 1500 mg daily of mycophenolate mofetil (MMF) was added to the corticosteroid therapy.

Serum creatinine levels decreased to 1.35 mg/dl and prednisolone dose was tapered monthly. However, 10 months later, serum creatinine levels increased again to 2.07 mg/dL (Fig. 2). ^67^Ga scintigraphy revealed significant radioactive tracer uptake in both kidneys (Fig. 1C), suggestive of AIN recurrence. Oral prednisolone (40 mg/d) was restarted, and valproate and lacosamide were discontinued based on a neurologist's recommendation. Renal function immediately improved, although minor epilepsy occurrence increased. Oral prednisolone was gradually tapered to 10 mg/d, after which serum creatinine levels increased again to 1.99 mg/dL. Repeat renal biopsy revealed similar findings of tubulitis with both lymphocytic and eosinophilic infiltrations. Prednisolone dose was increased to 60 mg (1.0 mg/kg) daily; nevertheless, it was difficult to discontinue carbamazepine due to the need for major epilepsy prevention. Since the patient had multiple AIN recurrence and was corticosteroid dependent, 1500 mg daily of oral mycophenolate mofetil (MMF) was added to the corticosteroid therapy. This treatment was well tolerated and enable successful prednisolone dose tapering to 5 mg daily, with serum creatinine level stabilization (Fig. 2). He experienced no AIN recurrence with MMF treatment for over 20 months.

## 3 Discussion

AIN is caused by a variety of disorders including allergic, infectious, and autoimmune diseases, and most frequently by drug allergies. Renal biopsy is useful for a definitive diagnosis of AIN by verifying the presence of “tubulitis” and determining the cell type of inflammatory infiltrate. Eosinophilic infiltration generally indicates that an allergic reaction is involved in the pathogenesis of AIN. However, eosinophilic infiltration can also be observed in some rare autoimmune disorders such as tubulointerstitial nephritis with uveitis (TINU), eosinophilic granulomatosis with polyangiitis, and Kimura's disease.^[4]^ The latter two diseases have specific clinical symptoms and can cause glomerular lesions; however, it may be difficult to distinguish TINU from DI-AIN. In patients with TINU, uveitis may follow renal involvement, and the renal pathology with eosinophilic infiltration in TINU is indistinguishable from that in DI-AIN, at least initially.^[5]^ TINU with late-onset uveitis is therefore probably misdiagnosed as DI-AIN if the patient takes one or more medications. In the present case, the patient was diagnosed with DI-AIN because of the lack of uveitis and involvement of other organs. Although serum levels of Krebs von den Lunge-6 were reported to specifically increase in patients with TINU,^[6]^ a subsequent larger study suggested that serum Krebs von den Lunge-6 was not different between patients with TINU and those with DI-AIN.^[7]^ Repeated ophthalmologic examination may be the only way to rule out TINU.

When DI-AIN is suspected, the putative offending drug(s) should be promptly discontinued. However, it may be difficult to identify the offending drug(s) due to polypharmacy. A recent study reported that 36.8% of elderly patients in the United States take >5 medications.^[8]^ Unfortunately, there are no definitive ways to select one or more offending drugs from many other drugs in patients with DI-AIN. Although the LST, an in vitro test for type IV allergic reactions, has been utilized to determine the offending drug(s) in patients with DI-AIN, it has an inadequate diagnostic sensitivity,^[9]^ and is therefore not widely recommended. Indeed, the patient in the present case experienced repeated AIN recurrence even after zoniamide discontinuation (as indicated by the positive LST result), suggesting that it was not the offending drug. Furthermore, it is usually difficult to discontinue “all” medications, since abrupt discontinuation of pivotal drugs such as anticoagulants and antiepileptics can lead to life-threatening events. Consequently, offending drugs may be continued if they cannot be replaced with appropriate alternatives.^[10]^ In the present case, recurrent eosinophilic interstitial nephritis might have been caused by the difficulty in discontinuing carbamazepine.

Recent studies have suggested the benefits of early corticosteroid therapy for DI-AIN^[11]^; most patients with DI-AIN experience no recurrence after corticosteroid therapy and discontinuation of the offending drug. However, in some cases, AIN recurs with steroid tapering, leading to corticosteroid dependency. As mentioned above, at least two factors contribute to corticosteroid dependency in eosinophilic interstitial nephritis: 1) the presence of underdetermined autoimmune processes, and 2) the difficulty in discontinuing the offending drug. Corticosteroid-dependent AIN is usually treated with repeated courses of corticosteroids to prevent disease progression. Although minimizing corticosteroid exposure is important for reducing adverse effects, there is little evidence regarding alternative management. MMF has been s therapeutic option for recurrent AIN caused by autoimmune disorders such as sarcoidosis and Sjögren's syndrome.^[12,13]^ In an earlier case series, eight patients with corticosteroid-dependent AIN underwent MMF treatment.^[14]^ This study included two patients with DI-AIN and MMF (1500 mg daily) successfully stabilized serum creatinine levels for over 25 months in both patients without complications. In a recent retrospective cohort study of 22 patients with recurrent AIN, 2 of 6 patients with DI-AIN underwent MMF treatment.^[3]^ One patient treated with a corticosteroid followed by MMF showed renal function improvement, whereas another with advanced renal failure showed no response to MMF treatment. These reports suggest a satisfactory tolerability and the steroid-sparing effect of MMF in corticosteroid-dependent DI-AIN. The present case experienced no obvious MMF-related adverse effects, including leukopenia and gastrointestinal symptoms. The scarcity of corticosteroid-dependent eosinophilic interstitial nephritis unfortunately makes it difficult to verify the efficacy of MMF in controlled trials. Although the benefits and risks require further verification in larger case studies, MMF may be considered as a candidate for the management of corticosteroid-dependent eosinophilic interstitial nephritis, with due attention to the common adverse effects.

## 4 Conclusion

It should be recognized that eosinophilic interstitial nephritis can recur even after corticosteroid therapy and discontinuation of the putative offending drug, leading to corticosteroid dependency. Undetermined autoimmune processes and polypharmacy contribute to corticosteroid dependency in patients with eosinophilic interstitial nephritis including DI-AIN. MMF may have a steroid-sparing effect under these conditions. However, the benefits and risks of MMF therapy for corticosteroid-dependent eosinophilic interstitial nephritis must be verified in larger case studies.

## Author contributions

**Conceptualization:** Katsuyuki Tanabe.

**Resources:** Katsuyuki Tanabe, Natsumi Matsuoka-Uchiyama, Tomoyo Mifune, Chieko Kawakita.

**Supervision:** Hitoshi Sugiyama, Jun Wada.

**Validation:** Katsuyuki Tanabe, Natsumi Matsuoka-Uchiyama, Hitoshi Sugiyama, Jun Wada.

**Visualization:** Katsuyuki Tanabe.

**Writing – original draft:** Katsuyuki Tanabe.
